# The formaldehyde stress on photosynthetic efficiency and oxidative stress response of moss *Racomitrium japonicum* L.

**DOI:** 10.3389/fpls.2024.1525522

**Published:** 2025-01-17

**Authors:** Wanting Li, Jiawen Zhang, Siqi Ma, Min Zhou, Ruixin Li, Hao Tang, Haiyan Qiu, Peng Ren, Yunlai Tang, Yunmei Lu, Renhua Huang, Ke Chen

**Affiliations:** ^1^ School of Life Science and Engineering, Southwest University of Science and Technology, Mianyang, Sichuan, China; ^2^ College of Biological Engineering, Jingchu University of Technology, Jingmen, Hubei, China; ^3^ Ecological Protection and Development Research Institute of Aba Tibetan and Qiang Autonomous Prefecture, Wenchuan, Sichuan, China; ^4^ Engineering Research Center of Biomass Materials, Ministry of Education, Southwest University of Science and Technology, Mianyang, Sichuan, China

**Keywords:** formaldehyde, photosynthesis, reactive oxygen species, antioxidant enzyme system, electron transport

## Abstract

**Introduction:**

Formaldehyde is a common gaseous pollutant emitted by buildings and decorative materials. In recent years, growing concerns have been raised regarding its harmful effects on health in indoor air. Therefore, this study aims to investigate the physiological and photosynthetic response mechanisms of *Racomitrium japonicum* under formaldehyde stress.

**Methods:**

*R. japonicum* was exposed to dynamic fumigation with formaldehyde for 7 days, with each day comprising an 8-h exposure period within a sealed container. The effects on plant structure, pigment content, photosynthetic efficiency, and reactive oxygen species (ROS) generation were assessed.

**Results and discussion:**

Our findings revealed that formaldehyde stress caused structural damage, reduced pigment content, decreased photosynthetic efficiency, and increased ROS production in *R. japonicum*. Significantly, distinct stress-response pathways were observed at different formaldehyde concentrations. In response to low and moderate formaldehyde concentrations, *R. japonicum* activated its antioxidant enzyme system to mitigate ROS accumulation. In contrast, the high-concentration treatment group demonstrated suppressed antioxidant enzyme activity. In response, *R. japonicum* used nonphotochemical quenching and activated cyclic electron flow to mitigate severe cellular damage. This study provides an in-depth understanding of the physiological changes in *R. japonicum* under formaldehyde stress, elucidating its response mechanisms. The findings offer valuable insights for developing effective indoor formaldehyde monitoring and purification methods.

## 1 Introduction

Formaldehyde, a common gaseous pollutant emitted from buildings and decorative materials (Adam et al., 2021; Hodgson et al., 2002), can cause irritation to the senses and lead to respiratory illnesses, immune system challenges, neurological disorders, and even develop causative connections with DNA adducts (Burgos-Barragan et al., 2017; McGregor et al., 2006; Vash, 2020). In 2017, the International Agency for Research on Cancer (IARC) of the World Health Organization classified formaldehyde as a group 1 carcinogen (WHO, 2020). Given its toxic effects, controlling formaldehyde is of utmost importance. Current techniques for removing formaldehyde from indoor environments include physical adsorption, chemical absorption, and catalytic and biological degradation (Peng et al., 2022). Among these methods, plant-based remediation remains the simplest, most environmentally friendly, and cost-effective alternative. Early studies by Wolverton (1984) suggest that plants could improve indoor air quality by removing organic pollutants. Further research has demonstrated that plant leaves can convert absorbed formaldehyde into carbon dioxide via the Calvin cycle or digest it into amino acids, lipids, free sugars, organic acids, and cell wall components (Kim et al., 2010; Salonen et al., 2009). In addition to entering the C1 metabolism through enzymatic reactions, plants can degrade formaldehyde via redox reactions. Liang et al. (2018) found that redox interactions between plant oxidants and formaldehyde may constitute the primary mechanism for formaldehyde decomposition in plants. They compared the formaldehyde dissipation capacity of fresh leaf extracts from *Chlorophytum comosum* (Liang et al., 2018). Several common ornamental plants, such as *C. comosum*, *Epipremnum aureum*, and *Nephrolepis exaltata*, and some small wild plants, including *Plantago asiatica*, *Taraxacum mongolicum*, and *Cremanthodium delavayi*, demonstrated outstanding capabilities in purifying air by absorbing and removing formaldehyde (Xu et al., 2011). Compared to other indoor plants, mosses and lichens offer greater potential for monitoringair pollutants, including formaldehyde, as they acquire all their nutrients and water from the air around them. However, comprehensive research on the response mechanisms of moss parameters to formaldehyde exposure is lacking.

Mosses, autotrophic higher plants that transitioned from aquatic to terrestrial habitats, derive their primary nutrients from substrates, rainwater, dew, and deposits of atmospheric dust (Bargagli, 2016). Due to their high sensitivity to environmental changes and adaptability to harsh conditions, mosses have been widely used for monitoring air quality and assessing environmental pollution (Liu et al., 2009; Ren et al., 2021; Xu et al., 2009). Moss leaves consist of a single or few layers of cells, lack a waxy layer, and have a large surface area. Some moss species have scales and papillae on their surfaces, which provide favorable conditions for the adsorption and retention of air pollutants (Chaligava et al., 2021; Gallego-Cartagena et al., 2021). In recent years, mosses and lichens have gained prominence as monitors and purifiers of pollutants, including heavy metals and radioactive nuclides (Kwon et al., 2021; Mahapatra et al., 2019; Stafilov et al., 2018). Mosses cultivated in harsh environments exhibit signs of damage, such as chlorophyll loss and leaf cell rupture. Changes in atmospheric pollutant composition can also affect photosynthesis, physiological metabolic pathways, secondary metabolites, and gene stability, leading to alterations in biological traits and physiological–biochemical parameters (Bargagli, 2016; Krommer et al., 2007).

Mosses possess distinctive physiological adaptation mechanisms that enable them to survive in challenging environments, such as high temperatures, extremely cold conditions, and drought, compared to other terrestrial plants. This makes them ideal for monitoring and validating atmospheric pollution (Sheng et al., 2022; Świsłowski et al., 2021). Due to their ornamental and economic value, moss plants are well-suited for short-term artificial cultivation and propagation. They offer easy management and a cost-effective approach to monitoring. Therefore, this study aims to investigate the photosynthetic and antioxidant physiological responses of *R. japonicum* under formaldehyde stress, potentially providing valuable insights into the adaptation mechanisms or biomonitor signals of moss in indoor air pollution.

## 2 Materials and methods

### 2.1 Plant materials and treatments

The moss (*R. japonicum*) utilized in this study was collected from Hanzhong, Shanxi, China. The selected *R. japonicum* samples were in a mature and healthy state after being thoroughly rinsed with deionized water to remove pebbles and weeds. They were then cultivated in a controlled greenhouse environment to simulate experimental conditions, including temperature, humidity, and light, for 2 weeks for acclimatization. The cultivation conditions included a diurnal temperature cycle of 25/20°C, light intensity of 2,000 lx, and relative humidity ranging from 60% to 70%. The moss samples were categorized into four treatment groups: control (0 mg m^−3^), low-concentration (10 mg m^−3^), moderate-concentration (50 mg m^−3^), and high-concentration (100 mg m^−3^). A gas collector was used to collect the gas inside the box, and the formaldehyde concentration inside the box according to the phenol reagent method (GBZ/T 300.99-2017, China). Following a 7-day cultivation period, *R. japonicum* was carefully transferred to glass culture dishes with an 11-cm diameter. The dishes were then placed inside a hermetically sealed fumigation chamber measuring 40 cm in length, width, and height, respectively. After closing the chamber, different volumes of formaldehyde were introduced, and the samples were subjected to an 8-h treatment each day, from 9:00 to 17:00, for 7 days. After the treatment, samples were collected to measure various photosynthetic and physiological parameters. At least three replicates for each parameter were measured to ensure reproducibility. The concentrations of formaldehyde were based on our pre-experiment results and previous work on formaldehyde stress in 15 ornamental plant species (Wang et al., 2020).

### 2.2 Morphological and structural analysis

Two to three roots of *R. japonicum*, treated for 7 days, were selected for photography. An appropriate amount of *R. japonicum* was taken, washed, and dried at 70°C until a constant weight was achieved. The samples were then ground into powder using liquid nitrogen and subjected to microstructure photography using a scanning electron microscope (SEM).

### 2.3 Determination of chlorophyll content

In this study, 0.3 g of *R. japonicum* was weighed, finely chopped, and placed into a 10-ml stoppered test tube. To facilitate extraction, 10 ml of a specific extraction solution consisting of a 1:1 ratio (v/v) mixture of acetone and ethanol was added to the test tube. The tube was securely covered and stored in a light-protected environment at 25°C for 24 h to allow for optimal extraction. After the moss plant leaves had completely discolored, indicating successful extraction, the contents of the test tubes were thoroughly mixed by shaking. Subsequently, 200 μl was withdrawn from the test tube, and the absorbance of the extracted solution was measured at two specific distinct wavelengths, 663 nm and 645 nm. This absorbance measurement served as a quantitative indicator of the desired compounds in the extraction solution.

### 2.4 Fluorescence imaging

Chlorophyll fluorescence imaging and related parameters of *R. japonicum* were measured using MINI-Pam (red LED version) and the imaging fluorometer software Win (Heinz Walz, Germany). Before measurement, the leaves were subjected to a 30-min dark treatment. The measurement light frequency was set to 1 Hz, intensity to 1 µmol m^−1^ s^−1^, photochemical intensity to 101 µmol m^−1^ s^−1^, saturation pulse intensity to 2,800 µmol m^−1^ s^−1^, the duration to 30 s, and the imaging area to 24 mm × 32 mm (Li et al., 2023).

### 2.5 Determination of fast chlorophyll fluorescence-induced kinetic curves

Chlorophyll fluorescence induction kinetics was rapidly assessed using a plant efficiency analyzer (M-PEA, Hansatech). When leaves were exposed to high-density photochemical light (10,000 μmol photons m^−2^ s^−1^), the fluorescence transient exhibited a multiphase rise with O-, J-, I-, and P-phases. This multiphase rise in fluorescence transients provides information regarding photosystem II (PSII) photochemistry, including electron transfer between the donor and acceptor sides of PSII. The four characteristic points on the OJIP curves (O, J, I, and P) correspond to time points of 0 ms, 2 ms, 30 ms, and 1,000 ms, respectively, with relative fluorescence intensities denoted by *F*_O_, *F*_J_, *F*_I_, and *F*_P_, respectively. The time points corresponding to 0.15 ms and 0.3 ms on the OJIP curve are designated as the L and K characteristic points, with corresponding relative fluorescence intensities denoted by *F*_L_ and *F*_K_, respectively (Tang et al., 2020). The OJIP curves for various treatments were normalized using O-P, O-J, and O-K-K. Normalization involved setting the relative fluorescence intensity at point O as 0 and at points P, J, and K as 1. The normalization equations used were *V*_O-P_ = (*F*_t_ – *F*_O_)/(*F*_p_ − *F*_O_), *V*_O-J_ = (*F*_t_ − *F*_O_)/(*F*_J_ − *F*_O_), and *V*_O-K_ = (*F*_t_ – *F*_O_)/(*F*_k_ – *F*_O_), where *F*_t_ represents the relative fluorescence intensity at different time points. The relative variable fluorescence intensities of the four characteristic points L, K, J, and I on the standardized curves are expressed as *V*_L_, *V*_K_, *V*_J_, and *V*_I_, respectively. Before the fluorescence measurements, all samples underwent a 30-min dark adaptation.

### 2.6 Rapid light-response curve determination

The measurement system used for simultaneous PSI and PSII measurements was the Dual-PAM 100 (Heinz Walz). To deplete the photosynthetic electron pool, plants were subjected to a 60-min dark adaptation. Subsequently, light at 500-μmol photons m^−2^ s^−1^ was applied for 5 min to activate the photosynthetic electron pool. The plants were then exposed to gradually increasing light intensities (0–1,181 mol photons m^−2^ s^−1^) to establish the light-response curves.

For PSI, the following parameters were calculated as follows: Y(I) = (*P*_m_′ − *P*)/*P*_m_; Y(ND) = *P*/*P*_m_; Y(NA) = (*P*_m_ − *P*_m_′)/*P*_m_, where Y(I) represents the photochemical quantum yield of PSI, reflecting the efficiency of PSII in converting absorbed light energy into chemical energy. Y(ND) represents the proportion of the oxidation state of PSI reaction center P700, and Y(NA) represents the proportion of the reduced state of PSI reaction center P700.

The PSII parameters were calculated as follows: Y(II) = (*F*_m_′/*F*_s_)/*F*_m_′, which is the actual light energy conversion efficiency of PSII under light conditions, indicating the performance of photosynthesis in the actual light environment; nonphotochemical quenching (NPQ) = (*F*_m_ − *F*_m_′)/*F*_m_′, which directly reflects the plant’s response and adaptation to light stress; Y(NO) = *F*_s_/*F*_m_, which reflects the photochemical energy conversion and protective regulation. Where *F*_m_ represents the maximum chlorophyll fluorescence intensity after dark adaptation. *F*_m_′ denotes the maximum chlorophyll fluorescence intensity after light adaptation. Fs are the steady-state fluorescence intensity following light acclimation. Y(II) denotes the effective photochemical quantum yield of PSII, NPQ is the non-photochemical quenching capacity of PSII, and Y(NO) denotes the non-regulatory heat dissipation of PSII.

The photosynthetic electron transfer rate was calculated using the following equation: ETRI = PPFD × Y(I) × 0. 84 × 0. 5; ETRII = PPFD × Y(II) × 0.84 × 0. 5, where ETRI and ETRII represent the electron transfer rates through PSI and PSII, respectively; ETRI-ETRII represents the cyclic electron transfer (CEF) rates; and PPFD represents the photosynthetic effective radiation intensity (Xia et al., 2022).

### 2.7 Determination of reactive oxygen content and staining localization

To determine hydrogen peroxide content, 0.1 g of the plant was taken and homogenized in liquid nitrogen with 1.7 ml of 0.1% TCA. The resulting homogenate was then centrifuged at 4°C for 15 min at 12,000 r/min. Subsequently, 1 ml of the supernatant was mixed with 0.2 ml of 1M KI and 0.1 ml of 0.1 M potassium phosphate buffer (pH 7.0). This mixture was allowed to react in the dark at room temperature for 1 h. The absorbance at 390 was measured using 0.1% TCA as a reference (Yesbergenova et al., 2005).

To determine superoxide anion content, 0.1 g of the plant was weighed and homogenized with 0.4 ml of 0.5 M potassium phosphate buffer (pH 7.4). The resulting homogenate was extracted for 10 min at 4°C and 3,500 r/min. Following this, 0.5 ml of the extract was mixed with 0.5 ml of 0.05 M potassium phosphate buffer (pH 7.8), and 1 ml of 1 mM hydroxylamine hydrochloride was added. The mixture was shaken well and then incubated at 25°C for 1 h. Subsequently, 1 ml of 17 mM *p*-aminobenzene sulfonic acid and 1 ml of 7 mM α-naphthylamine were added sequentially, mixed with rapid shaking, and incubated for an additional 1 h at 25°C. The mixture was quickly mixed and shaken well after adding 1 ml of 17 mM *p*-aminobenzenesulfonic acid and 1 ml of 7 mM α-naphthylamine (Yergaliyev et al., 2016). Subsequently, the samples were kept at 25°C for 20 min, followed by centrifugation at 3,000 r/min for 3 min. The pink aqueous phase was then measured at 530 nm and zeroed with distilled water. The rate of superoxide anion production was determined from the NO_2_^−^ standard curve and fresh weight (FW).

To determine malondialdehyde (MDA) content, 0.2 g of the plant was weighed and extracted in two batches using a 10% TCA solution, bringing the final volume to 2 ml. The extraction was centrifuged at 3,000 r/min for 10 min, and the resulting supernatant was retained for measurement. In the color reaction step, 1 ml of the supernatant and 2 ml of a 0.6% thiobarbituric acid solution were combined in a stoppered test tube. The mixture was well-mixed, covered, and placed in a boiling water bath for 20 min to develop the color. After rapid cooling, the solution was centrifuged (Srivastava et al., 2017). The absorbance values at 532 nm, 600 nm, and 450 nm were then determined for the supernatants.

Hydrogen peroxide-positioned staining using the diaminobenzidine method: a 50 ml of 1 mg/ml 3,3-diaminobenzidine (DAB) solution was adjusted to a pH of 3.0 with 0.2 M HCI. To this solution, 25 μl of Tween-20 (0.05% V) and 2.5 ml of 200 mM Na_2_HPO_4_ were added. Due to the light-sensitive nature of DAB, precautions were taken to shield it from light (Achary et al., 2008). The moss plants subjected to treatment were fully immersed in the DAB staining solution and placed in a shaker at 80–100 rpm, 25°C, for 4 h, shielded from light using tin foil. After staining, the plants were bleached (anhydrous ethanol [V]: acetone [V] = 1:1) in a boiling water bath for 15 min using a bleach solution.

Superoxide anion staining-nitrotetrazolium chloride blue method: A total of 0.05 g of nitrotetrazolium chloride blue (nitroblue tetrazolium [NBT]) was weighed and dissolved in 50 mM phosphate buffer (pH 7.5) to prepare 50 ml of 0.1% NBT staining solution (Rao and Davis, 1999). The moss plants were immersed in the staining solution and incubated in the dark at 25°C for 12 h. The color was then removed in two steps using a bleaching solution (acetone [V] + anhydrous ethanol [V] = 1:1 and anhydrous ethanol [V]: glacial acetic acid [V]: glycerol [V] = 3:1:1].

### 2.8 Determination of antioxidant enzyme system activity

Superoxide dismutase (SOD), peroxidase (POD), and catalase (CAT) levels were measured using kits manufactured by the Nanjing Jianjian Bioengineering Institute (Nanjing Jiancheng Bioengineering Institute, Nanjing, China), while ascorbate peroxidase (APX), dehydroascorbate reductase (DHAR), and monodehydroascorbate reductase (MDHAR) levels were measured using kits manufactured by Solepol (Solarbio Life Sciences, Beijing, China).

### 2.9 Statistical analysis

Data were presented as the means ± SE of independent measurements. Group differences were evaluated using one-way analysis of variance (ANOVA), followed by Duncan’s new multiple range test at a significance level of *p* < 0.05, conducted using SPSS 18.0. Basic data visualization and processing were performed using Origin 2018 software.

## 3 Results

### 3.1 Morphological and structural effects of formaldehyde stress on *R. japonicum*

**Figure 1:**
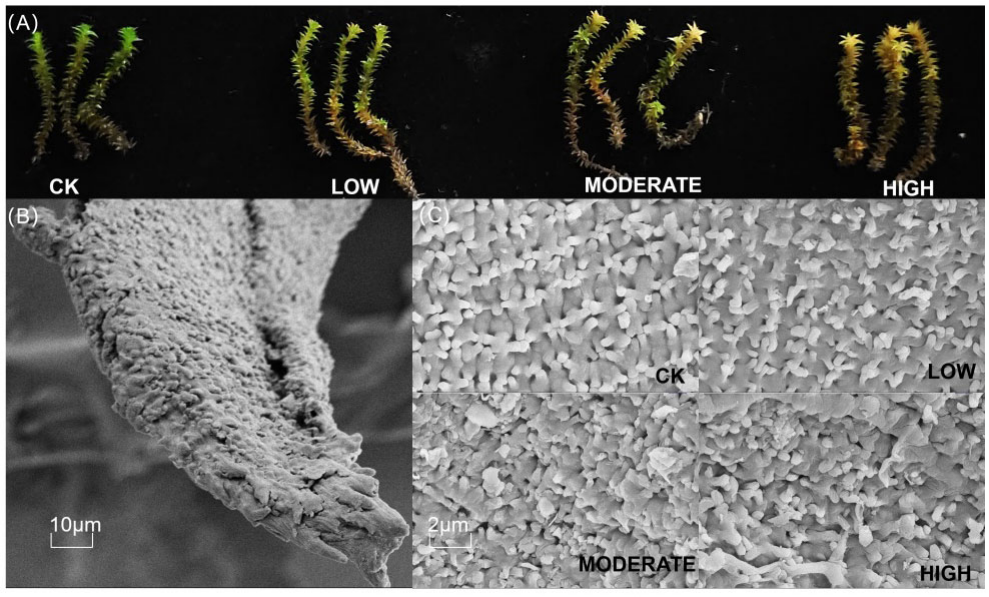
Morphological changes **(A)** and microstructural changes **(C)** in *R. japonicum* after formaldehyde stress. **(B)** The scanning electron microscope images of control leaves at 10 μm.

Formaldehyde fumigation partially disrupted the morphology of *R. japonicum*. Formaldehyde caused the green color of *R. japonicum* leaves to transition to yellow, with the degree of yellowing positively correlated with formaldehyde concentration (**Figure 1A**). At low concentrations, yellowing was initially observed at the leaf tips, but as the concentration increased, it gradually spread downward along the leaves. All parts of *R. japonicum* in the high-concentration group displayed yellow hues. To understand the changes in the *R. japonicum* microstructure, we used an SEM. In the control group, the moss exhibited a well-organized surface structure with uniform interstices. In contrast, the moss in the low-concentration group showed a gradual thinning of the surface structure. As the formaldehyde concentration increased in the medium- and high-concentration groups, the disruption of the moss surface structure increased, leading to larger gaps and a more dispersed distribution (**Figure 1C**).

### 3.2 Effect of formaldehyde stress on chlorophyll content and fluorescence imaging of the *R. japonicum*

When plants are exposed to external environmental stress, chlorophyll content serves as an effective indicator of photosynthetic damage. The low-concentration group exhibited a of 19.7% reduction in chlorophyll *a* content and a 23.4% decrease in chlorophyll *b* content compared to the control group. Similarly, the moderate-concentration group showed a significant decline of 55.9% in chlorophyll *a* content and a 35.1% decrease in chlorophyll *b* content. Significantly, the high-concentration group experienced a substantial 68.9% decrease in chlorophyll *a* content and a 54.2% reduction in chlorophyll *b* content (**Table 1**). These findings highlight the heightened sensitivity of *R. japonicum* chlorophyll content to formaldehyde exposure. Furthermore, in the moderate- and high-concentration groups, the chlorophyll *a*/*b* ratios were decreased by 31.3% and 32.1%, respectively, compared to the control group. In contrast, the low-concentration group showed no significant changes.

**Table 1:** Chlorophyll content of *Racomitrium japonicum* after formaldehyde stress.

	Chl *a* (μg·mg^−1^)	Chl *b* (μg·mg^−1^)	Chl *a*/Chl *b*
CK	0.177 ± 0.001 a	0.094 ± 0.001 a	1.877 ± 0.024 a
Low	0.142 ± 0.003 b	0.072 ± 0.002 b	1.983 ± 0.001 a
Moderate	0.078 ± 0.003 c	0.060 ± 0.002 c	1.289 ± 0.086 b
High	0.055 ± 0.001 d	0.043 ± 0.002 d	1.274 ± 0.067 b

Values are expressed as means ± SE (*n* = 3). Different letters represent significant differences between the treatments (*p* < 0.05).

**Figure 2:**
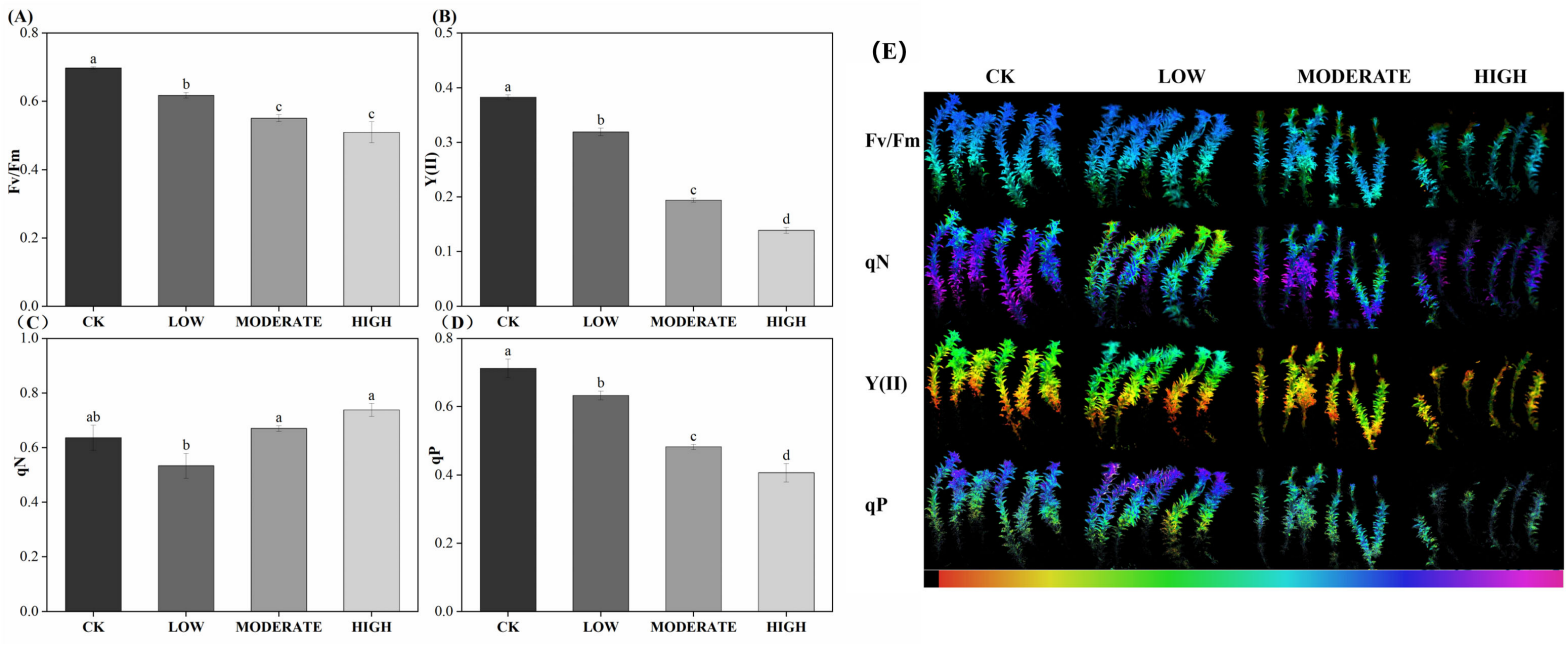
Chlorophyll fluorescence parameters **(A–D)** and imaging **(E)** of *R. japonicum* under various formaldehyde concentrations. The false color code depicted at the bottom of each image ranges from 0.000 (black) to 1.000 (pink). Values are expressed as means ± SE (*n* = 3). Different letters represent significant differences between treatments (*p* < 0.05).

Chlorophyll fluorescence imaging of *R. japonicum* (**Figure 2**) and chlorophyll fluorescence parameters (**Table 2**) provided valuable insights into the effects of formaldehyde stress on photosynthesis. *F*_v_/*F*_m_, a commonly utilized and frequently measured parameter in chlorophyll fluorescence analysis, serves as an indicator of the maximum photosynthetic efficiency of photosystem II, reflecting the plant’s ability to convert light energy optimally. After exposure to varying formaldehyde concentrations, the *F*_v_/*F*_m_ values of *R. japonicum* decreased significantly. Specifically, the low-, moderate-, and high-concentration groups exhibited a decrease of 11.4%, 21%, and 27.4%, respectively, compared to the control group. Y(II), representing the actual photosynthetic efficiency of photosystem II, accurately represents the current light-energy conversion efficiency of the photosynthetic apparatus. Under formaldehyde stress, Y(II) in *R. japonicum* significantly declined with increasing formaldehyde concentrations (*p* < 0.05). Fluorescence quenching can occur due to enhanced photosynthetic activity or increased thermal dissipation during photosynthesis. Photochemical quenching (qP) refers to fluorescence quenching resulting from photosynthesis. In contrast, nonphotochemical quenching (qN) describes fluorescence quenching arising from thermal dissipation. Compared to the control group, qP decreased by 11%, 32.3%, and 42.9% in the low-, moderate-, and high-concentration groups, respectively, indicating a decrease in photosynthetic activity under formaldehyde stress. Moreover, compared with the low-concentration group, qN increased by 20% and 38.4% in the moderate- and high-concentration groups, respectively.

**Table 2:** Parameters for fitting the light-response curve of *R. japonicum* under different concentrations of formaldehyde stress.

	Parameters of RLCs of ETR(I)			Parameters of RLCs of ETR(II)		
	*α* (*e* ^−^photon*^−^* ^1^)	ETRmax (µmol *e^−^* m*^−^* ^2^ s*^−^* ^1^)	*I*k (µmol photon m*^−^* ^2^ s*^−^* ^1^)	*α* (*e^−^* photon*^−^* ^1^)	ETRmax (µmol *e^−^* m*^−^* ^2^ s*^−^* ^1^)	*I*k (µmol photon m*^−^* ^2^ s*^−^* ^1^)
CK	0.468 ± 0.020 a	169.060 ± 17.865 a	360.897 ± 49.196 ab	0.509 ± 0.038 a	94.586 ± 12.756 a	185.747 ± 35.171 a
Low	0.476 ± 0.024 a	117.235 ± 6.271 b	246.240 ± 20.551 b	0.574 ± 0.078 a	64.010 ± 2.895 b	111.489 ± 29.975 ab
Moderate	0.462 ± 0.028 a	101.664 ± 7.627 b	219.854 ± 28.491 b	0.680 ± 0.119 a	37.148 ± 3.180 c	54.596 ± 9.827 b
High	0.292 ± 0.035 b	129.340 ± 18.447 ab	442.499 ± 93.426 a	0.223 ± 0.054 b	21.973 ± 5.897 c	98.489 ± 23.746 ab

Values are expressed as means ± SE (*n* = 3). Different letters represent significant differences between the treatments (*p* < 0.05).

### 3.3 Fast-phase fluorescence of chlorophyll

**Figure 3:**
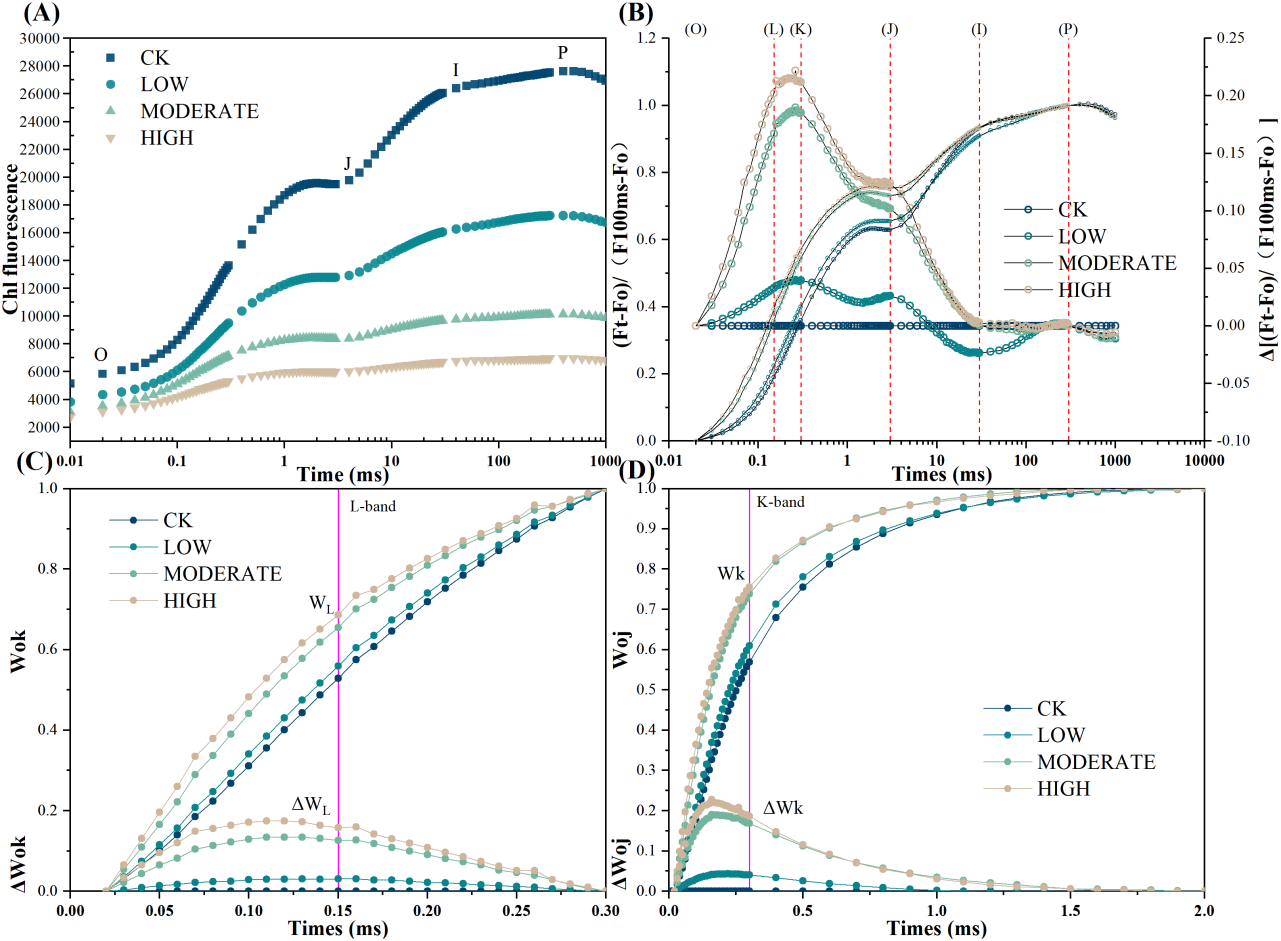
Chlorophyll fast-phase fluorescence of *R. japonicum* under varied formaldehyde concentrations. **(A)** Raw Chl *a* fluorescence rise kinetics showing different concentrations of formaldehyde stress on a logarithmic time scale. **(B)** Chl *a* fluorescence rise kinetics normalized by *F*_O_ and *F*_M_ as *V*_t_ = (*F*_t_ − *F*_O_)/(*F*_M_ − *F*_O_) in a logarithmic time scale. Δ*V*_t_ = *V*_t_ (treatment) − *V*_t_ (control). **(C)** Fluorescence rise kinetics normalized by *F*_O_ and *F*_K_ as WOK = (*F*_t_ − *F*_O_)/(*F*_K_ − *F*_O_), and the difference kinetics Δ*W*_OK_ = *W*_OK_ (treatment) − *W*_OK_ (control) in a linear time scale from 0 to 300 μs. **(D)** Fluorescence rise kinetics normalized by *F*_O_ and *F*_J_ as *W*_OJ_ = (*F*_t_ − *F*_O_)/(*F*_J_ − *F*_O_), with the difference kinetics Δ*W*_OJ_ = *W*_OJ_ (treatment) − *W*_OJ_ (control) in a linear time scale from 0 to 2 ms.

To better understand the changes occurring in photosystem II of *R. japonicum* under formaldehyde-induced stress, we analyzed the rapid chlorophyll fluorescence induction kinetics curve, commonly referred to as the OJIP curve (**Figure 3A**). The O point on the curve indicates the initial fluorescence. In contrast, the P point represents the peak fluorescence intensity. Following exposure to formaldehyde, the O and P points decreased their values, corresponding to increasing formaldehyde concentrations. To standardize the OJIP curve, it was normalized to the *F*_m_-*F*_o_ baseline. Subsequently, *V*_t_ was calculated by subtracting the kinetics of the treated group from those of the control group (**Figure 3B**). Comparative analysis revealed distinct variations in the formaldehyde-treated samples compared to the control group. Specifically, the appearance of L and K peaks was observed, with the L peak attaining its highest magnitude in the high-concentration group (**Figures 3C, D**). The K peak is caused by the dissociation of the oxygen-evolving complex from the Mn complex, which inhibits the supply of electrons from water splitting to the electron donor of PSII. This results in an imbalance in the ability to supply electrons to the PSII reaction center and the ability to transfer electrons away from the PSII reaction center. Compared to the control group, the L peak appeared under formaldehyde treatment, with the highest intensity observed in the high-concentration group. This suggests that formaldehyde exposure leads to the dissociation of the antenna pigment complex, further damaging the integrity of the thylakoid membrane in *R. japonicum*. The highest points of the L and K peaks at high concentrations indicate that formaldehyde stress destroyed the photosynthetic apparatus of the moss, with more severe damage observed at higher concentrations.

### 3.4 Effect of formaldehyde stress on the rapid light-response curve of *R. japonicum*

**Figure 4:**
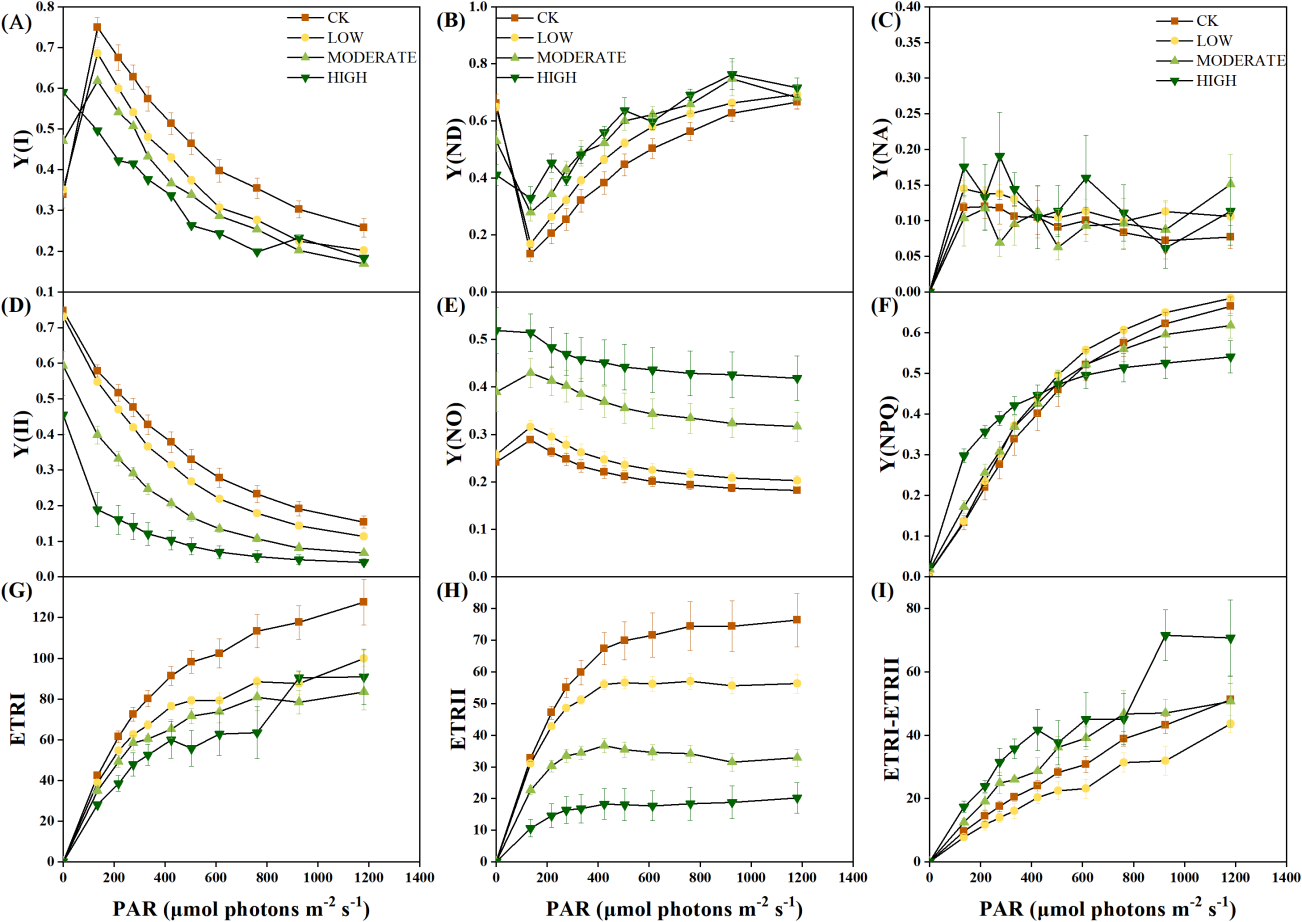
Rapid light-response curve of *R. japonicum* under varied formaldehyde stress concentrations. **(A)** Y(I), **(B)** Y(ND), **(C)** Y(NA), **(D)** Y(II), **(E)** Y(NO), **(F)** Y(NPQ), **(G)** ETRI, **(H)** ETRII, and **(I)** ETRI-ETRII. Values are expressed as means ± SE (*n* = 3).

The rapid light-response curve provides real-time insights into the physiological status of photosynthesis. Y(I) represents the actual photochemical efficiency of photosystem I (PSI). The PSI photochemical quantum yield Y(I) decreased with increasing light intensity (**Figure 4A**). Starting at an illumination intensity of 135 μmol photons m^−2^ s^−1^, the middle- and high-concentration groups exhibited significantly lower Y(I) values than the control group (*p* < 0.05). This indicates a reduction in the actual photochemical efficiency of PSI in *R. japonicum* under moderate to high formaldehyde stress, leading to the functional impairment of PSI. Y(ND) represents the quantum yield of nonphotochemical dissipation caused by the donor-side limitation of PSI (**Figure 4B**). From an illumination intensity of 135 μmol photons m^−2^ s^−1^, the Y(ND) values in the middle- and high-concentration groups were significantly higher than those in the control group (*p* < 0.05), suggesting an elevation in donor-side limitation of PSI under moderate to high formaldehyde stress. Y(NA) represents the quantum yield of nonphotochemical dissipation caused by acceptor-side limitation in PSI. The values in all groups were relatively low and showed no significant changes (*p* > 0.05) (**Figure 4C**). Y(II) represents the actual photochemical efficiency of PSII. The middle- and high-concentration groups showed significantly lower Y(II) values than the control group (*p* < 0.05), suggesting that moderate to high formaldehyde stress restricts the functionality of PSII (**Figure 4D**). Y(NO) represents the quantum yield of nonregulated energy dissipation in PSII and serves as an indicator of photodamage. The Y(NO) values in the intermediate and high formaldehyde concentration groups were significantly higher than those in the control group (*p* < 0.05), suggesting that moderate-to-high formaldehyde stress caused a certain degree of photodamage to the photosynthetic apparatus (**Figure 4E**). Y(NPQ) represents the energy absorbed by PSII, which dissipates as heat and serves as a photoprotection indicator (**Figure 4F**). At an illumination intensity of 135–275 μmol photons m^−2^ s^−1^, the high concentration group exhibited significantly higher Y(NPQ) values compared to the other treatment groups (*p* < 0.05), suggesting the activation of thermal dissipation as a protective response to formaldehyde stress under low light conditions. However, at an illumination intensity of 1,181 μmol photons m^−2^ s^−1^, the high-concentration group showed significantly lower Y(NPQ) values than the control and low-concentration groups (*p* < 0.05). This decrease could be attributed to the high light intensity, potentially limiting the thermal dissipation mechanism in *R. japonicum.*

ETRI represents the relative linear electron flow through photosystem I, whereas ETRII denotes the relative linear electron flow through photosystem II. The middle- and high-concentration groups demonstrated significantly lower ETRI and ETRII values than the control group (*p* < 0.05) (**Figures 4G, H**), suggesting that formaldehyde stress concurrently impaired the photosynthetic efficiency of both PSII and PSI. The difference between ETRI and ETRII is known as CEF. At an illumination intensity of 135–424 μmol photons m^−2^ s^−1^, the high-concentration group exhibited significantly higher CEF values than the control and low-concentration groups (*p* < 0.05), indicating the activation of CEF in response to high formaldehyde concentration to alleviate photodamage to PSI (**Figure 4I**). A rectangular hyperbolic model was used for nonlinear fitting to quantitatively describe the rapid light-response curve. The fitted parameters (**Table 2**) included α, which represents the initial slope of the rapid light-response curve and reflects the efficiency of the photosynthetic organs in utilizing light energy. Under high formaldehyde concentration treatment, the initial slope of the rapid light-response curve in *R. japonicum* significantly decreased (*p* < 0.05), indicating a significant reduction in the efficiency of light energy utilization by the photosynthetic organs. ETRmax represents the potential maximum relative electron transport rate derived from the fitting. The ETRIImax values in the low-, middle-, and high-concentration groups were significantly lower than those in the control group (*p* < 0.05), suggesting that formaldehyde stress affects electron transport in *R. japonicum*, with PSII being the most susceptible to formaldehyde. Additionally, the ETRIImax values in the low- and middle-concentration groups were significantly lower than those in the control group (*p <*0.05), indicating impairment of the electron transport capacity in PSI caused by moderate to low formaldehyde concentrations. However, no significant difference was observed between the high-concentration and control groups (*p* > 0.05). Point Ik, representing the intersection of the initial slope line with the ETRmax level on the *x*-axis, reflects the tolerance of the sample to high light intensity. No significant difference was observed in PSII between the high-concentration and other treatment groups (*p* > 0.05). In contrast, for PSI, the high-concentration group showed a significant increase than the low- and medium-concentration groups (*p* < 0.05). This increase may be because of the activation of cyclic electron flow around PSI, thereby enhancing the tolerance of the plant to high light intensity.

### 3.5 Changes in antioxidant physiology of *R. japonicum* under formaldehyde stress

**Figure 5:**
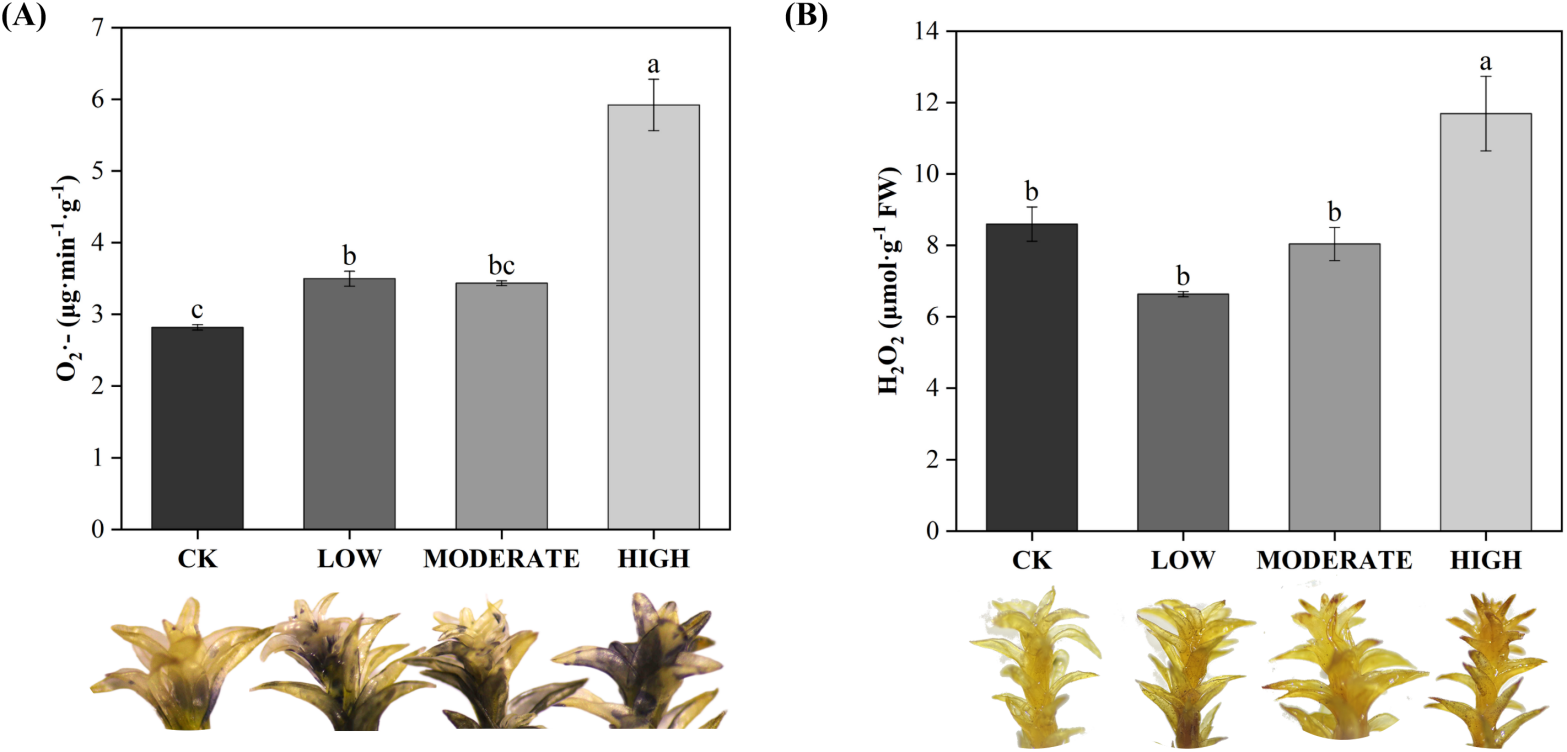
Effects of different concentrations of formaldehyde stress on the content of reactive oxygen species in *R. japonicum*. **(A)** Superoxide anion content and leaf staining in *R. japonicum*. **(B)** Hydrogen peroxide content and leaf staining in *R. japonicum*. Values are expressed as means ± SE (*n* = 3). Different letters represent significant differences between treatments (*p* < 0.05).

**Figure 6:**
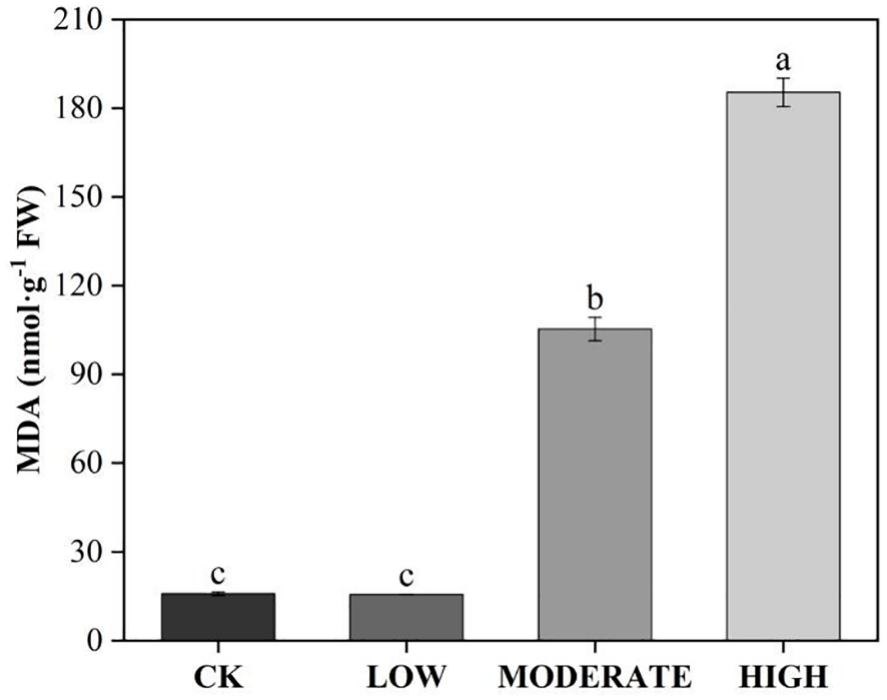
Effects of different concentrations of formaldehyde stress on the MDA content of *R. japonicum*. Values are expressed as means ± SE (*n* = 3). Different letters represent significant differences between treatments (*p* < 0.05).

To analyze the effect of formaldehyde stress on oxidative damage to the photosystems of *R. japonicum*, we measured the accumulation of ROS. The treatment groups demonstrated elevated levels of superoxide anion (O_2_^·−^), hydrogen peroxide (H_2_O_2_), and MDA compared to the control group (**Figures 5A, B**, **6**). Specifically, the low-concentration group showed a 24.1% increase in O_2_^·−^ accumulation compared to the control group. Additionally, the MDA content in the medium-concentration group was 6.6 times higher than that in the control group. The accumulation of ROS in *R. japonicum* was most pronounced under high formaldehyde stress, exhibiting 2.1, 1.36, and 11.7 times higher levels of O_2_^·−^, H_2_O_2_, and MDA, respectively, compared to the control group. Tissue staining with NBT for superoxide anions revealed purple staining in the *R. japonicum* leaves after formaldehyde treatment (**Figure 5A**), with the highest intensity and distribution observed in the high-concentration group. Additionally, tissue staining with DAB for hydrogen peroxide showed brown deposits at the tips of *R. japonicum* leaves under formaldehyde treatment (**Figure 5B**), with both intensity and the number of staining sites increasing with higher formaldehyde concentrations. These findings indicate that the accumulation of ROS in *R. japonicum* gradually increases with higher formaldehyde concentrations, suggesting that the presence of formaldehyde can induce oxidative damage in the plant.

**Figure 7:**
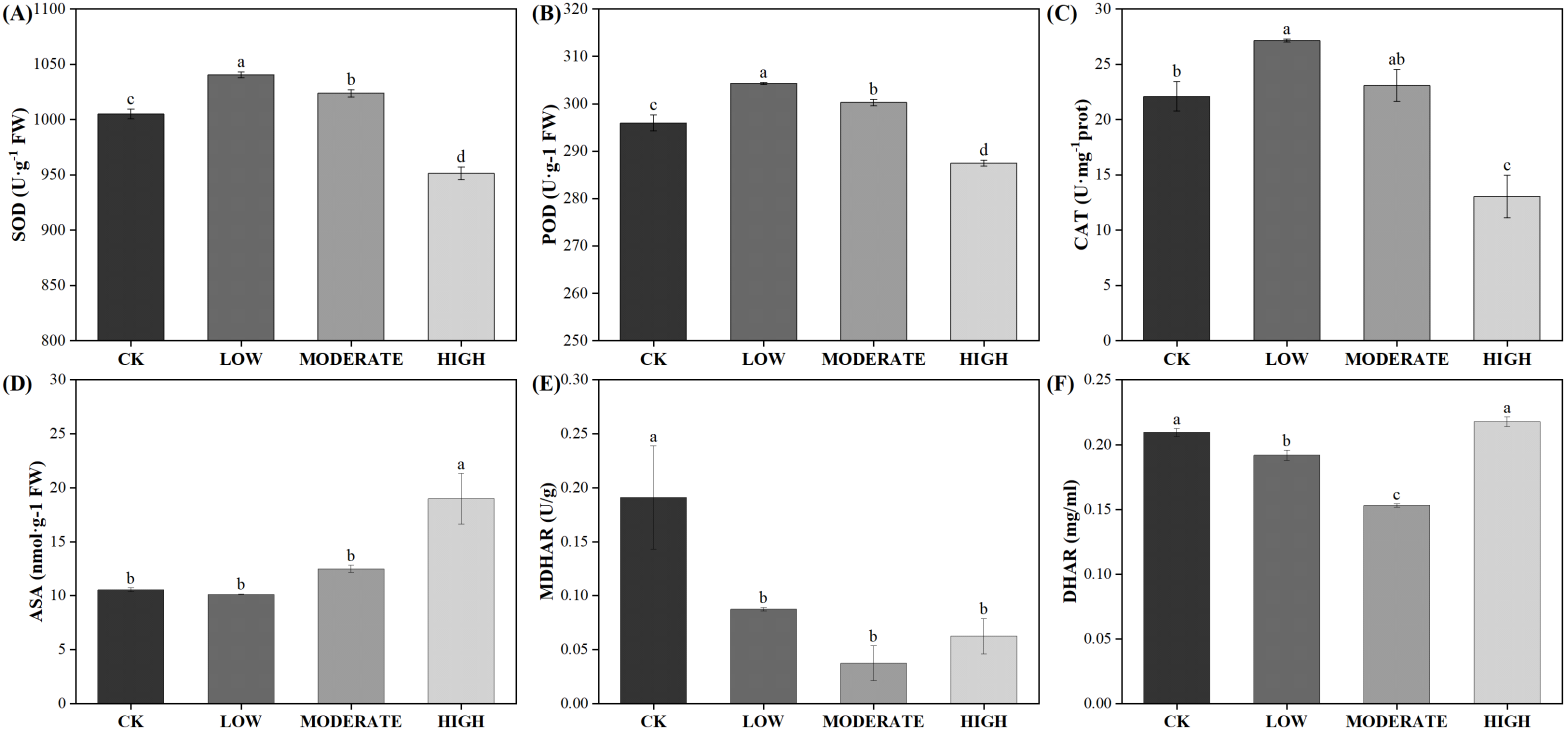
Effects of varied concentrations of formaldehyde stress on the antioxidant system of *R. japonicum*. **(A)** SOD, **(B)** POD, **(C)** CAT, **(D)** ASA, **(E)** MDHAR, and **(F)** DHAR. Values are expressed as means ± SE (*n* = 3). Different letters represent significant differences between treatments (p < 0.05).

Following oxidative damage, plants activate various protective mechanisms, with the antioxidant enzyme system being the key mechanism for ROS clearance. Under low-concentration formaldehyde stress, the activities of SOD, POD, and CAT increased significantly (*p* < 0.05) (**Figures 7A–C**). Under moderate formaldehyde treatment, SOD and POD activities significantly increased (*p* < 0.05). In contrast, CAT showed no significant difference compared with the control group (*p* > 0.05). However, under high formaldehyde stress, the activities of SOD, POD, and CAT decreased significantly (*p* < 0.05). High-stress conditions may lead to changes in certain chemicals within the plant body, which could directly affect the active sites of antioxidant enzymes, thereby reducing their catalytic efficiency. The damage to plant cell membranes caused by high formaldehyde stress affects the function and activity of membrane-related enzymes (including antioxidant enzymes). This indicates that the antioxidant enzyme system can effectively function within a certain range of formaldehyde concentrations to clear ROS. However, its activity decreases at high formaldehyde concentrations. Apart from the antioxidant enzyme system, nonenzymatic antioxidants also play a role in ROS clearance. In the ascorbate-glutathione cycle, the activities of MDHAR and DHAR in the low-concentration group were reduced by 54.2% and 8.42%, respectively, compared to the control group. In the moderate-concentration group, MDHAR and DHAR activities were reduced by 80.2% and 26.9%, respectively, compared to the control group. In the high-concentration group, the activities of APX and MDHAR were reduced by 61.3% and 67.1%, respectively, compared to the control group (**Figures 7D–F**).

## 4 Discussion

### 4.1 Effects of formaldehyde stress on the morphological structure of *R. japonicum*

Scanning electron microscopy revealed the emergence of protrusions on the surface of *R. japonicum* (**Figure 1B**), which enhanced the surface contact area of the leaves and promoted the adsorption of substances from the air. The plant’s morphology serves as a strong indicator of the air quality in its surrounding environment. After exposure to moderate and high concentrations of formaldehyde, *R. japonicum* leaves exhibited wilting and a color change from green to yellow. Scanning electron microscopy revealed a transition in the microstructure of *R. japonicum*, from a dense and orderly arrangement to a disordered state. Previous studies have shown that 15 plant species exhibited various forms of morphological damage, such as leaf surface damage, tip withering, and leaf wilting, after exposure to different concentrations of formaldehyde fumigation. The degree of damage can serve as an indicator of the plant’s resistance level (Wang et al., 2020), which aligns with our findings that the pore structure of *R. japonicum* leaves was moderately damaged by formaldehyde stress. By utilizing this characteristic of moss, we can explore several possible indoor air quality management strategies, such as designing and developing moss-based air purification devices that take advantage of moss’s natural absorption capacity to remove formaldehyde from indoor air. Using moss as an indoor decorative element, such as wall coverings or hanging decorations, cannot only beautify the indoor environment but also purify the air simultaneously. Creating moss gardens or small ecosystems indoors by planting various moss species can further enhance air purification. Additionally, a new type of air purification material has been developed by combining moss with biological filter media. This material can be placed in indoor ventilation systems to effectively remove pollutants, such as formaldehyde, from the air through the absorption and conversion processes facilitated by moss.

### 4.2 Effect of formaldehyde stress on chlorophyll content and fluorescence of *R. japonicum*

Changes in the pigment content within chloroplasts usually directly affect the photosynthetic efficiency of plants. Nonbiological stressors can alter the structure and content of chlorophyll, disrupt its function, and, consequently, affect photosynthesis. Previous studies have indicated that under formaldehyde stress, plants experience inhibited photosynthesis and a reduction in chlorophyll content (Li et al., 2019; Wang et al., 2020). Our findings align with this pattern, revealing a significant decrease in chlorophyll content in *R. japonicum* following formaldehyde stress. This suggests that formaldehyde impacts plant chlorophyll synthesis. Chlorophyll *a*, a critical light-harvesting pigment, is present in reduced levels, leading to diminished light-harvesting pigment content in *R. japonicum*. This reduction affects the efficiency of light utilization, consequently impairing plant photosynthesis. Given that approximately 90% of Chl *b* is associated with the light-harvesting chlorophyll *a*/*b* protein complex of PSII (LHCII), the reduction in Chl *b* suggests the degradation of LHCII (Horie et al., 2009). Furthermore, our results indicate that the chlorophyll content in *R. japonicum* was more sensitive to formaldehyde than chlorophyll *b*. This may explain the significant decrease in the chlorophyll *a*/*b* ratio at moderate to high formaldehyde concentrations compared to the control group (*p* < 0.05). Our study indicates that the suppression of pigment synthesis results in a decrease in photosynthetic activity.

Chlorophyll fluorescence serves as a classical indicator of photosynthesis, offering a nondestructive and rapid assessment of plant photosynthetic activity. As formaldehyde concentrations increased, both *F*_v_/*F*_m_ and Y(II) decreased, highlighting the significant impact of formaldehyde on the photosynthetic efficiency of *R. japonicum* PSII and a reduction in its overall photosynthetic activity. Photochemical quenching (qP) reflects plant photosynthetic activity. In contrast, nonphotochemical quenching (qN) reflects the plant’s ability to dissipate excess light energy as heat, serving as a measure of photoprotective capacity. As formaldehyde concentration increased, qP decreased, and qN increased. This indicates that under moderate to high formaldehyde stress, the photosynthetic activity of *R. japonicum* is inhibited, prompting the plant to activate photoprotective mechanisms for dissipate excess light energy and protect the photosynthetic apparatus (Ren et al., 2022). In conclusion, formaldehyde stress affected chlorophyll synthesis and disrupted photosynthesis in *R. japonicum* in a concentration-dependent manner.

### 4.3 Effect of formaldehyde stress on fast-phase chlorophyll fluorescence in *R. japonicum*

Although previous studies have reported the uptake of pollutants, including heavy metals, organic chemicals, PAHs, PM10, and PM2.5 particles, in mosses for air quality biomonitoring (Varela et al., 2023), our study first reported chlorophyll OJIP fluorescence responses of selected mosses to formaldehyde stress. Chlorophyll OJIP fluorescence kinetics analysis, a nondestructive detection method widely utilized to assess the impact of nonbiological stress on plant photosynthetic metabolism (Goltsev et al., 2016), was used to assess the effects of formaldehyde stress on photosystem II in *R. japonicum*. A more detailed understanding of the damage to photosystem II was achieved through chlorophyll fast fluorescence. In this analysis, point O represents the initial fluorescence, and its intensity correlates with the content of the antenna pigments and the activity of the reaction center. The initial fluorescence decreased as the formaldehyde concentration increased, possibly due to a decrease in chlorophyll content in *R. japonicum* compared to the control group. Point P represents the maximum fluorescence, and its magnitude is related to the structure and function of the antenna, as well as the extent of energy dissipation. As the formaldehyde concentration increased, the maximum fluorescence decreased compared to the control group, possibly due to damage to the photosynthetic antenna. Through double normalization of the OJIP curve and individual stage analyses, the emergence of L and K peaks was identified after formaldehyde treatment. Compared to the control group, the appearance of the L peak was observed in the formaldehyde treatment group, with the highest L peak observed in the high-concentration group. These findings indicate that the presence of formaldehyde led to dissociation of the antenna pigment complex, resulting in further damage to *R. japonicum* thylakoid membrane integrity (**Figure 3C**). The K step length (300 μs) in the chlorophyll fluorescence transient serves as an indicator of damage to the oxygen-evolving complex (OEC) in the photosynthetic apparatus. The K step length in the treatment group was significantly higher than that in the control group (*p* < 0.05) (**Figure 3D**), indicating the inhibition of donor-side PSII activity and damage to the OEC. The peaks of the L and K signals were most prominent at high formaldehyde concentrations, indicating degradation of the photosynthetic apparatus in *R. japonicum* under formaldehyde stress, with more severe damage observed at higher concentrations.

### 4.4 Effect of formaldehyde stress on the rapid light-response curve of *R. japonicum*

The Dual-PAM-100 system, offering simultaneous measurements of chlorophyll fluorescence and P700+ absorbance, provides a valuable tool for assessing changes in both PSII and PSI. As formaldehyde concentrations increased, Y(I), Y(II), ETR(I), and ETR(II) showed decreasing trends, whereas Y(ND) and Y(NO) showed increasing trends. The increase in Y(NO) indicates an elevated proportion of closed PSII centers and the inability of reaction centers to cope with excessive light under high levels of formaldehyde stress. Formaldehyde stress reduced the quantum yields of PSI and PSII, as well as the relative linear electron flow rate, while imposing constraints on the donor side of PSI. The significant increase in Y(ND) and the relatively modest alteration in Y(NA) suggest that the decrease in the quantum yield of PSI was primarily due to limitations on its donor side. Y(ND) represents the fraction of P700 oxidized due to a deficiency of donors or electron deprivation in PSI. The small antenna size in PSII effectively limits the photochemistry of PSI by inefficiently absorbing light and encountering donor-side limitations (Huang et al., 2010). The significant Y(ND) increase under formaldehyde stress suggests that PSI remains well-regulated even after formaldehyde treatment. This decrease in PSI photochemistry was due to insufficient electron donors and light absorption by PSI (Deng et al., 2013). A decrease in Y(I) indicates significant impairment of photosynthetic function (Bailey et al., 2008). The concurrent reduction in Y(I) and Y(II) suggests that formaldehyde stress inhibited electron transfer in both PSII and PSI of moss. The decrease in ETRmax(II) and ETRmax(I) (**Table 2**) and the increase in Y(ND) (**Figure 4B**) confirm this inhibitory effect.

As the formaldehyde concentration increased, a significant rise in the rate of CEF was observed compared to the control, indicating the activation of CEF under formaldehyde stress. CEF around PSI mitigates damage from excess electron flow to PSI by generating ΔpH (Ren et al., 2022). ΔpH plays an important role in regulating photosynthesis by modulating the light-harvesting efficiency of antennae through the qE mechanism, facilitating the dissipation of excess light energy. The generation of ΔpH also slows down the electron transfer from PSII to PSI through Cytb6f, preventing over-reduction of the PSI reaction center and reducing the production of superoxide and singlet oxygen in the thylakoid membrane (Takagi et al., 2017). ΔpH serves as a central signal, stabilizing the oxygen-evolving complex and activating NPQ (Tan et al., 2020; Tikkanen and Aro, 2014). Activation of CEF and NPQ is an important mechanism for protecting the photosynthetic apparatus under formaldehyde stress. Various abiotic stresses, including high temperature, high light, and low temperature, can reduce the ability of plants to absorb and utilize light, leading to the accumulation of excess light energy in the photosystem. This, in turn, influences photosystem activity and reduces photosynthetic efficiency (Faik et al., 2016; Zhou et al., 2018). To prevent excessive damage to photosystems, plants may dissipate the excess energy accumulated in PSII and PSI centers through mechanisms such as NPQ, the xanthophyll cycle, state transitions, and alternative electron pathways (Lu et al., 2020; Pinnola and Bassi, 2018).

### 4.5 Effects of formaldehyde stress on the antioxidant physiology of *R. japonicum*

**Figure 8:**
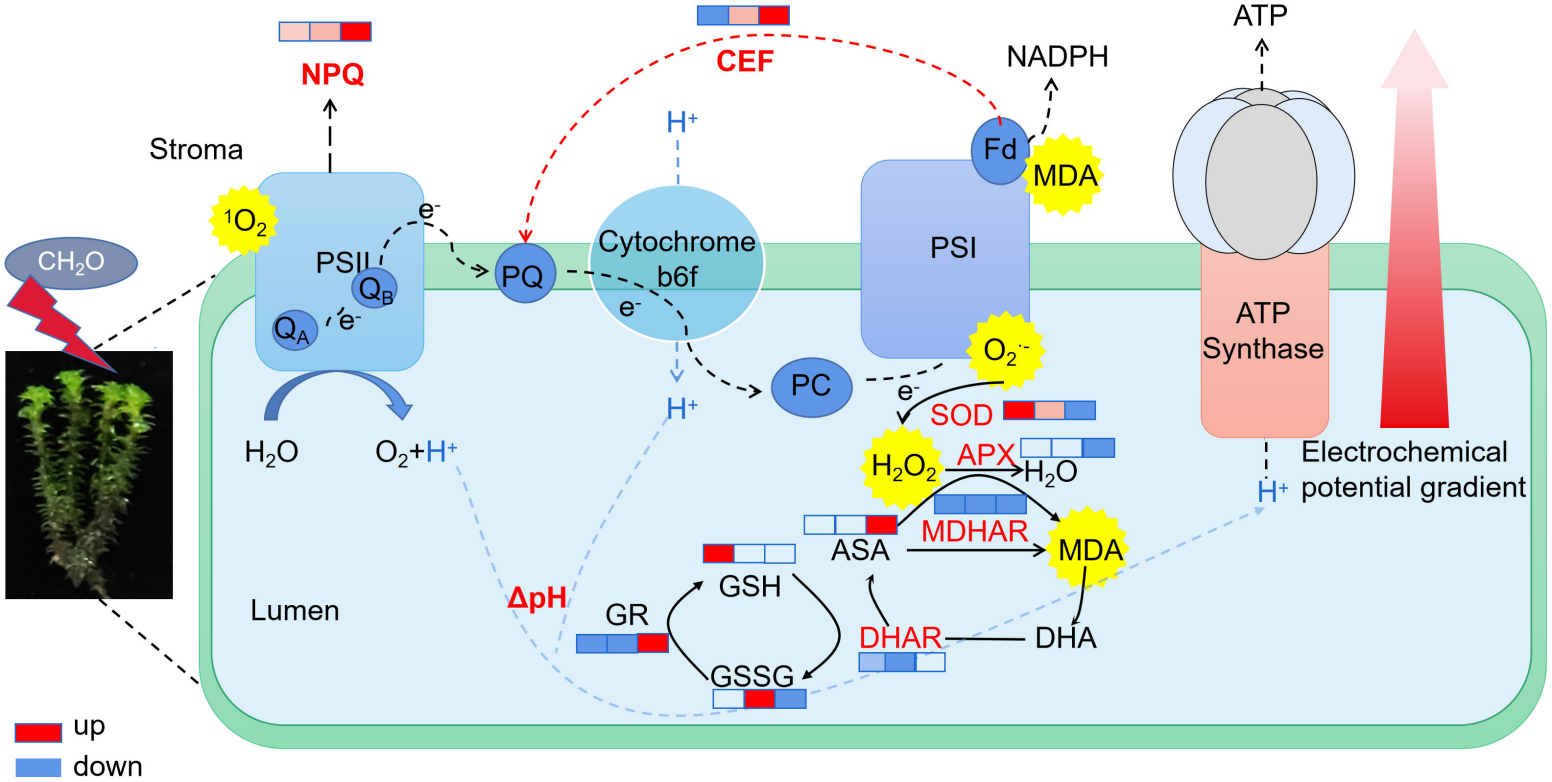
Electron transport and antioxidant physiological changes in *R. japonicum* under various formaldehyde concentrations. The boxes in the figure, from left to right, represent the low, moderate, and high-concentration groups. The colors in the boxes indicate changes compared to the control group, with red representing upregulation and blue representing downregulation. The shade of the color indicates the degree of change.

In stressful conditions, a reduction in light utilization efficiency leads to an increase in excess excitation energy, resulting in the generation of ROS and the accumulation of reduced power within PSI (Chovancek et al., 2019; Lu et al., 2017). If alternative electron flows do not quench excess electrons in PSI, ROS are generated in PSI, causing damage. ROS comprise a group of oxygen-derived reduction products with high redox potentials, including singlet oxygen (^1^O), superoxide anion (O_2_^·−^), hydroxyl radical (·OH), and hydrogen peroxide (H_2_O_2_). ROS are prevalent throughout plants, with endogenous ROS primarily stem from nonphotosynthetic pathways in mitochondria and peroxisomes, as well as photosynthetic metabolism in chloroplasts. The photosynthetic pathway in plants is particularly sensitive to stress and serves as a major source of ROS (Takagi et al., 2017). Our results are consistent with the idea that plants maintain a dynamic balance between ROS production and clearance by regulating enzyme-mediated antioxidant systems and synthesizing nonenzymatic antioxidants (Bobrovskikh et al., 2020; Zhanassova et al., 2021). The antioxidant enzymes directly scavenge ROS and are extensively involved in plant oxidative stress responses. Formaldehyde induces the production of superoxide anions in *R. japonicum*, typically originating from chloroplast photosystems I and II. As shown in **Figure 8**, this suggests that under formaldehyde stress, the light quantum absorbed by photosynthetic pigments exceeds the energy required for *R. japonicum* to fix CO_2_, potentially leading to the production of superoxide anions in chloroplasts due to excess light energy. The increased SOD activity promotes the conversion of superoxide anions into hydrogen peroxide, while increased POD and CAT activities facilitate the conversion of hydrogen peroxide into water, thereby preventing the accumulation of excessive hydrogen peroxide and cellular oxidative damage. These findings indicate that *R. japonicum* can experience disturbances in intracellular ROS levels at low to moderate formaldehyde concentrations. However, the response of intracellular antioxidant enzymes effectively prevents oxidative damage caused by ROS accumulation. High-concentration stress may also trigger changes in signal transduction pathways within plants, which can regulate the synthesis or degradation of antioxidant enzymes, thereby affecting enzyme activity. MDA serves as a reliable indicator of membrane damage resulting from oxidative stress, particularly due to membrane lipid peroxidation (Peng et al., 2022). When *R. japonicum* is exposed to formaldehyde concentrations (< 50 mg/m^3^), it activates the antioxidant enzyme system to effectively eliminate ROS within cells. The generation and clearance of ROS reached a dynamic balance, thereby protecting the cells. However, at higher formaldehyde concentrations (100 mg/m^3^), the activities of the three enzymes in the antioxidant system significantly decreased (*p* < 0.05). ROS accumulate within cells without proper clearance, leading to membrane lipid peroxidation and increased MDA content. The formaldehyde stress may also damage to other biomolecules within the cell (such as proteins and DNA), which could indirectly affect the activity of antioxidant enzymes, as their function often requires interaction with other biomolecules.

## 5 Conclusions

From both photosynthetic and antioxidant physiological perspectives, the response mechanisms of *R. japonicum* under different concentrations of formaldehyde stress were systematically elucidated. The revealed that the formaldehyde stress caused structural damage, reduced pigment content, decreased photosynthetic efficiency, and increased ROS production in *R. japonicum*. In response to formaldehyde stress (< 50 mg/m^3^), *R. japonicum* activated its antioxidant enzyme system to mitigate ROS accumulation. However, in the high-concentration treatment (100 mg/m^3^), antioxidant enzyme activity was suppressed, likely due to high stress directly affecting the active sites of the enzymes, thereby reducing their catalytic efficiency. As a result, *R. japonicum* used nonphotochemical quenching and activated cyclic electron flow to mitigate severe cellular damage. However, to gain a more comprehensive understanding of photosynthetic efficiency and the potential real-world applications of moss for monitor formaldehyde, further studies are needed to select additional moss species across various concentrations and evaluate their CO_2_ absorption rates in formaldehyde-polluted environments.

## Data Availability

The raw data supporting the conclusions of this article will be made available by the authors, without undue reservation.
